# Defining type 2 asthma and patients eligible for dupilumab in Italy: a biomarker-based analysis

**DOI:** 10.1186/s12948-021-00146-9

**Published:** 2021-05-21

**Authors:** Giorgio Walter Canonica, Francesco Blasi, Nunzio Crimi, Pierluigi Paggiaro, Alberto Papi, Francesca Fanelli, Annalisa Stassaldi, Gianluca Furneri

**Affiliations:** 1 Personalized Medicine, Asthma and Allergy, Humanitas Clinical and Research Center IRCCS, Via Manzoni 56, 20089 Rozzano, MI Italy; 2 Department of Biomedical Sciences, Humanitas University, Pieve Emanuele, MI Italy; 3 Fondazione IRCCS Ca Granda Ospedale Maggiore Policlinico, Internal Medicine Department, Respiratory Unit and Cystic Fibrosis Adult Center and Department of Pathophysiology and Transplantation, University of Milan, Milan, Italy; 4 Respiratory Medicine Unit, A.O.U. Policlinico-Vittorio Emanuele, Catania, Italy; 5 Department of Clinical and Experimental Medicine, University of Catania, Catania, Italy; 6 Department of Surgery, Medicine, Molecular Biology and Critical Care, University of Pisa, Pisa, Italy; 7 Respiratory Medicine Unit, Department of Medical Sciences, University of Ferrara, Ferrara, Italy; 8 Sanofi S.P.A., Milan, Italy; 9 EBMA Consulting S.R.L., Via per Carpiano 2, 20077 Melegnano, MI Italy

**Keywords:** Type 2 asthma, Dupilumab, Biomarkers, Italy

## Abstract

**Background:**

Asthma is a chronic disease characterized by airway hyperresponsiveness, inflammation and mucus production. In Type 2 asthma, two phenotypic components are often co-expressed (eosinophilic and allergic). Elevated biomarker levels, such as eosinophils (EOS), fraction of exhaled nitric oxide (FeNO) and immunoglobulin E (IgE), are key clinical indicators of Type 2 inflammation. Dupilumab has been recently approved for the treatment of uncontrolled severe Type 2 asthma. Type 2 asthma includes allergic and/or eosinophilic phenotypes. The aim of this analysis was to estimate the dupilumab-eligible population in Italy and characterize it by expected biomarker status.

**Methods:**

A 4-step approach was carried out to calculate dupilumab-eligible population. The approach consisted in: (1) estimating the total number of asthma patients in Italy (using 2016–2017 Italian-adapted Global Initiative for Asthma -GINA- guidelines); (2) estimating the number of severe asthma patients with poorly controlled or uncontrolled disease (using the findings of two recent administrative claim analyses conducted in Italy); (3) stratifying the severe uncontrolled population by biomarker levels (EOS, FeNO and IgE) according to the outcomes of the QUEST trial (a clinical study assessing the efficacy of dupilumab in patients with uncontrolled moderate-to-severe asthma; NCT02414854); (4) identifying the sub-populations of severe uncontrolled asthma patients characterised by raised blood EOS and/or FeNO level (thus indicated to receive dupilumab).

**Results:**

According to these estimates, about 3.3 million asthmatic patients live in Italy (6.10% of the population). Of them, almost 20 thousand (N = 19,960) have uncontrolled severe asthma. Dupilumab-eligible patients would be N = 15,988, corresponding to 80.1% of the total uncontrolled severe population. Most of these patients (89.3%; N = 14,271) have at least an increase of EOS level, while slightly more than half (51.9%; N = 8,303) have raised levels of both biomarkers. Increased FeNO levels without increased EOS are observed less frequently (N = 1,717; 10.7% of the eligible population).

**Conclusions:**

There is a strong rationale for testing all asthma biomarkers during diagnosis and disease follow-up. Given the large availability and the limited costs, these tests are cost-effective tools to detect severe Type 2 asthma, stratify patients by phenotype, and drive appropriate treatment decisions.

**Supplementary Information:**

The online version contains supplementary material available at 10.1186/s12948-021-00146-9.

## Introduction

Asthma is a chronic disease affecting people of any age characterized by airway hyperresponsiveness (AHR), airway obstruction and inflammation, intermittent airflow and mucus production [1, 2]. Wheezing, cough, chest tightness, and shortness of breath, usually accompanied by airflow limitation, are the most typical symptoms of asthma patients [1]. Asthma is also a common condition: recent estimates report that about 300 million people have asthma worldwide, with prevalence of the disease likely increasing by a further 100 million by 2025 [3–6].

The way asthma has been defined and classified has changed over time. In the past, asthma was considered “a single entity” disease, characterized by abnormal response of T-helper cell Type 2 (Th2) cells and B cells [7]. Today, the term “asthma” is rather intended as a collection of several distinct endotypes (T2-high vs T2-low) and phenotypes (e.g. young atopic, obese middle aged, smokers, late onset, etc.) that can lead to different symptomatology and variable level of airflow obstruction [7]. Particularly in severe asthma, in-depth understanding of asthma pathogenesis and pathophysiology, together with identification of phenotypes, has important implications on treatment decision making.

The pathophysiology of asthma is characterized by the immune response of two CD4 + T-cell subsets, Th1 and Th2 cells. Specifically, the Type 2 asthma endotype (in the past known as “T2-high”) is originated from the complex mechanisms of Type 2 inflammation where two phenotypic components are co-expressed (eosinophilic, allergic, or mixed). Type 2 inflammation is mainly driven by Th2 cell activation, which triggers an abnormal generation of cytokines (interleukin IL-4, IL-5 and IL-13), in response to the detection of different agents (e.g. allergens, pollution, viruses, etc.) [1, 7, 8]. Indeed, further research in the last decades clarified that the underlying mechanism of Type 2 inflammation is much more complex, being mediated by many other players, such as innate lymphoid cells (ILC2s), produced in response to external agents [1, 7, 8]. Th2 and ILC2-derived IL-4, IL-5, and IL-13 generate the typical pathophysiological effects of asthma, including activation and recruitment of eosinophils in the airways, IgE production by activated plasma cells, AHR and airway remodelling.

**Fig. 1:**
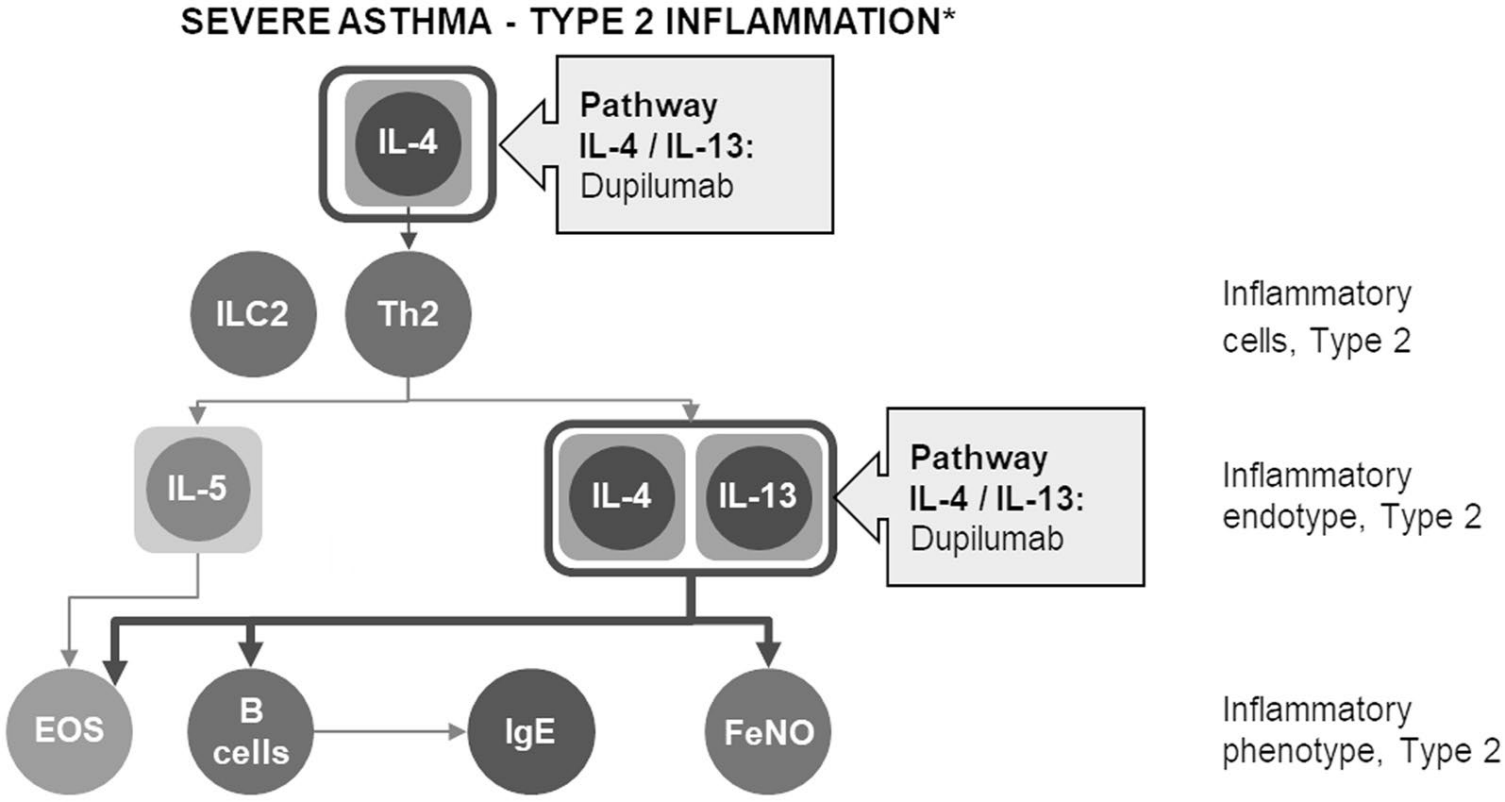
Pathophysiology of Type 2 inflammation in severe asthma. IL-4 /IL-13 pathway. IL-5 pathway

IL-4 and IL-13 are relatively upstream players in the inflammatory cascade, and they are responsible for various pro-inflammatory activities. IL-13 stimulates the production of eotaxin 1 in airway inflammatory cells, causes airway smooth muscle and goblet cell hyperplasia, transforms fibroblasts into myofibroblasts, increases tracheal-bronchial mucus secretion, collagen production by fibroblasts and subepithelial basal membrane thickening, which are all features of airway remodelling [9]. IL-4 plays a central role in the activation of the whole cytokine cascade in Type 2 inflammation. It acts on mast cells, producing IL-4/13 and IL-5 themselves. Furthermore, IL-4/13 induce isotype class switching of B cells to produce IgE, and directly affect the lung airway structure. In particular, IL-4/13 induce basement membrane thickening, impairment of epithelial integrity and proliferation of M2 macrophages, which directly changes the lung airway structure (e.g. fibrosis). Finally, IL-13 promotes inducible nitric oxide (iNO)-synthase activity and nitric oxide (NO) production, increasing the fraction of exhaled nitric oxide (FeNO) level (Fig. 1).

Type 2 is probably the most common type of asthma: about 50%–70% of people with asthma have an underlying Type 2 inflammation [8, 10, 11]. From a diagnosis standpoint, blood eosinophils (EOS) count has been commonly used as a biomarker to identify Type 2 asthma. Indeed, other biomarkers, such serum immunoglobulin-E (IgE) levels, and more recently FeNO, have been linked to mechanisms involved in Type 2 inflammation [1, 12, 13]. More specifically, FeNO has emerged as an important biomarker, as it informs about the inflammatory state of the airways [9, 14]. Therefore, it has been effectively used as predictive biomarker in several clinical trials evaluating the effectiveness of biological therapies in moderate-severe asthma [15–18]. Furthermore, during the process of allergic inflammation, FeNO is produced by the airway epithelium in excessive amount, because of the nitric oxide synthase upregulation, so elevated FeNO is a good index of Type 2 inflammation.

Elevated biomarker levels, such as EOS, FeNO and IgE, are key clinical indicators of Type 2 inflammation [11, 19]. EOS level is mediated by IL-4, IL-13, and IL-5 and defines eosinophilic asthma [20, 21]. FeNO level correlates with serum IgE and sputum EOS; it is mediated by IL-4 and IL-13 [22], and correlates also with allergic asthma [20, 21]. Type 2 inflammation is the key driver of all Type 2 asthma phenotypes, including eosinophilic, allergic or mixed (i.e. eosinophilic and allergic are co-expressed) [21].

Today, clinical guidelines recommend testing severe asthma patients for multiple biomarkers, i.e. blood and sputum EOS, FeNO, IgE (thresholds: blood EOS ≥ 150 cells/μL; FeNO ≥ 20 ppb; IgE ≥ 30 IU/mL) especially in presence of refractory disease with underlying Type 2 inflammation [1]. There is a clear rationale for investigating these biomarkers simultaneously: while in many patients more than one biomarker can be overexpressed, others could have only one increased, independently one from the other [7]. In this latter case, some biomarker expression might have been suppressed by pharmacological treatment, (i.e. oral corticosteroids for blood eosinophils) but asthma would remain clinically uncontrolled [1, 23].

Evaluation of Type 2 inflammation biomarkers is of critical importance to guide treatment decision in severe asthma which is refractory to conventional medical therapy. The standard of care of asthma is based on inhaled corticosteroids (ICS), either as monotherapy or in combination with other treatments, such as long-acting β2-adrenoreceptor agonists (LABAs) and/or cysteinyl-leukotriene type 1 receptor antagonists (LTRAs) [1, 23]. However, treatment is estimated to be ineffective in around 5%–10% of the overall population (i.e. severe patients), who then need escalation to high dose ICS and/or systemic therapy with corticosteroids, associated with uncertain clinical response, high risk of adverse events and long-term contraindications [1, 23, 24]. It is estimated that asthma is not adequately controlled in about half of this severe, refractory population [25]. Furthermore, the clinical picture can be complicated by coexisting Type 2 inflammatory conditions that are commonly observed in these patients: chronic rhinitis and sinusitis, nasal polyps, atopic dermatitis [26].

Over the last 15–20 years, several biologic therapies have been developed to address the unmet medical need in severe asthma, and many others are under evaluation in clinical trials [27]. Treatment with biologic therapies has improved the prognosis of patients with uncontrolled severe asthma, and concomitantly, the understanding of the complex pathogenesis and pathophysiology mechanisms of the disease, favouring the concept of patient stratification by biomarker [27]. Among biologics, dupilumab (Dupixent), a fully human monoclonal antibody directed against the alpha subunit of the IL-4 receptor (and inhibiting IL-4 and IL-13 signalling) has been shown to be safe and effective in adolescents and adults with severe uncontrolled asthma [28–30]. Recently, dupilumab has been authorized by the European Medicines Agency (EMA) for the *“treatment of adults and adolescents 12 years and older as add-on maintenance treatment for severe asthma with Type 2 inflammation, characterised by raised blood EOS and/or raised FeNO”* [31]. Therefore, dupilumab is the first biologic approved and specifically indicated for the treatment of uncontrolled severe asthma with Type 2 inflammation: asthma that includes allergic (anti-IgE) and/or eosinophilic (anti-IL5) phenotypes. Instead, the other biologic drugs approved by the EMA are indicated for specific Type 2 severe asthma phenotypes (e.g. “Allergic” for anti-IgE or “Eosinophilic” for anti-IL5) [32–36]. The rationale for this biomarker-related indication comes from the QUEST study, a 52-week placebo-controlled, phase 3 confirmatory study (NCT02414854) enrolling patients aged ≥ 12 years, one of the largest ever-conducted trials characterizing patients in terms of biomarker expression (EOS, FeNO, IgE).

Stratification of patient population by biomarkers to identify the right eligible patients is a crucial task in the “biologic-era”. While personalized treatment of asthma is producing significant benefits for patients, asthma management costs are increasing. Notably, about 50% of the global asthma budget is allocated to severe patients (who account for < 10% of the overall population) [37, 38].

The need for managing resources appropriately and controlling therapeutic expenditure makes biomarker testing even more important, for budget allocation purposes and cost-effective use of high-cost drugs, such as biologics. Quite recently, many studies have been conducted with the objective of estimating the epidemiological and clinical burden of severe asthma in Italy [39–41]. In this paper we aimed to: (1) estimate the number of Type 2 severe asthma patients who would be eligible for dupilumab treatment in Italy, according to its approved indication; and (2) characterize the dupilumab-eligible population by expected biomarker status.

## Methods

This paper aims to calculate the number of Type 2 severe asthma patients who would be eligible for dupilumab treatment in Italy.

**Fig. 2:**
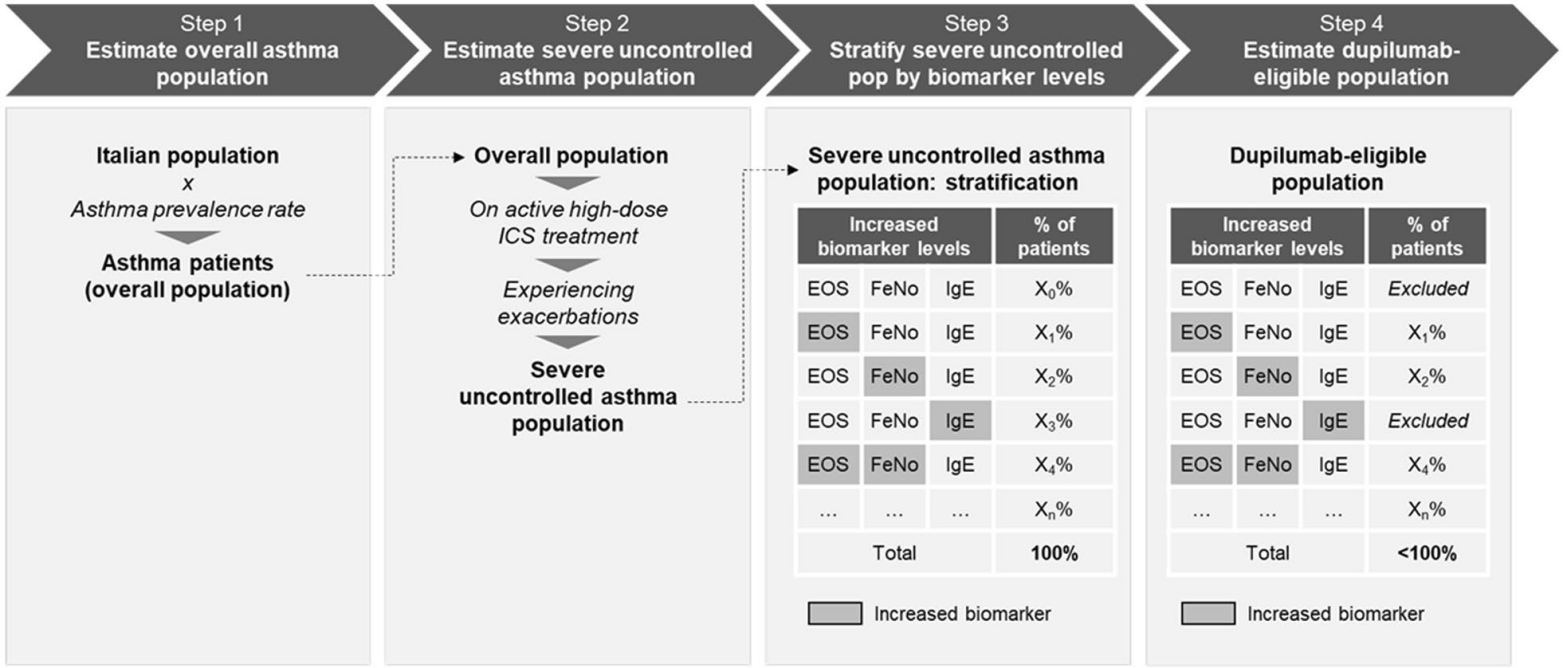
Methodological approach to estimate dupilumab-eligible population in Italy (illustrative)

A 4-step approach was used: (1) estimation of the total number of asthma patients (overall asthma population); (2) estimation of the number of severe asthma patients, who are poorly controlled or uncontrolled (despite ICS treatment;); (3) stratification of the severe uncontrolled asthma population, by biomarker levels; (4) identification and estimation of the sub-populations of severe uncontrolled asthma patients eligible for treatment with dupilumab, based on appropriate biomarker levels. The methodological approach flow is shown in Fig. 2.

### Data sources

#### Step 1

To estimate the number of patients affected by asthma in Italy, a prevalence rate of 6.1% was used. This estimate was gathered from the 2016–2017 Italian-adapted GINA (Global Initiative for Asthma) guidelines [23], and refers to a recent study conducted by general practitioners in Italy, according to which 61 out of 1000 subjects aged > 15 years old were presenting some form of asthma. In this study, prevalence rates were slightly higher for women (6.6%) than for men (5.5%) and tended to decrease with older age. This prevalence rate was considered appropriate to inform the analysis, because it was referred to a mixed population of adolescents and adults (i.e. patients aged > 15), which is within the dupilumab approved indication (i.e. treatment of patients aged 12 and older). This prevalence rate was also consistent with GEIRD study estimates (prevalence rate of 6.6% in the Italian population aged 20–44 years old) [42]. The prevalence rate was then multiplied by the Italian population, aged ≥ 12 years (source: Italian Institute of Statistics, ISTAT [43]).

#### Step 2

The second step of the analysis was to extract the population with severe uncontrolled asthma from the overall patient population with asthma. For this purpose, two different sources were used and then compared to each other: (1) a recent publication from Pedrini et al., 2017 [39]; (2) the analysis conducted by Region Veneto in 2016 (source: regional guidelines for pharmacological management of severe uncontrolled asthma [41]). The aim of both of these analyses was to identify and estimate patients with severe uncontrolled asthma, requiring follow-up in specialized hospital centers. In both cases, the analysis was conducted using Italian administrative claim databases as main data source.

In their study, Pedrini et al. retrospectively analysed administrative data concerning adult patients (≥ 18 years old) registered in an Italian healthcare system database (the Accounting and Reporting Console -ARCo- database) [39]. Over the 2013–2014 time period, patients with severe refractory asthma were identified through: (1) prescription of omalizumab (anatomical therapeutic chemical -ATC- code: R03DX05) or; (2) asthma exemption code (007.493; it allows asthma patients to avoid participating in the cost of health services in Italy) associated with a prescription of high-dose systemic corticosteroids identifying asthma exacerbations (prednisone 25 mg, ATC code: H02AB07; or methylprednisolone 16 mg; ATC code: H02AB04); or (3) asthma exemption code (007.493) associated with outpatient service/day hospital with injection of other therapeutic or prophylactic substances (ICD9 CM procedure code: 99.29). With this approach, the authors estimated an overall prevalence of severe refractory asthma of 0.04%.

The regional analysis conducted in Veneto adopted stricter requirements (compared with Pedrini et al.) to identify more appropriately patients with severe refractory asthma requiring regular specialist monitoring [41]. The analysis progressively filtered asthma patients through the following inclusion criteria: (1) asthma exemption code; (2) spirometry execution; (3) active pharmacological treatment with ICS + LABA, and/or theophylline and/or leukotriene receptor antagonists; (4) high-dose ICS treatment (prescription of ICS maximum dose); (5) high-rate adherence (level of annual coverage ≥ 80%); (6) disease exacerbations (≥ 2 episodes/year of inpatient admission or treatment with systemic corticosteroids for > 3 days in the ambulatory setting). The codes considered for patient inclusion are reported in Additional File 1: TableS3. With this approach, the authors estimated an overall prevalence of severe refractory asthma of 0.034%. The “patient-funnel” approach defined in this publication is shown in Table 1.

**Table 1:** Estimation of patients with severe uncontrolled asthma in Italy

#	Group of subjects	Estimation	Number of subjects (N)	Source
1	Italian population, ≥ 12 years	–	5,41,19,490	ISTAT 2019 [43]
2	Patients with asthma	6.10% of #1	33,01,289	[41]
3	Patients with asthma exemption code	36.29% of #2	11,97,821	
4	Patients with spirometry (last 12 months)	27.74% of #3	3,32,281	
5	Patients treated with ICS	46.95% of #4	1,55,997	
6	Patients treated with high-dose ICS	43.05% of #5	67,151	
7	Patients treated with high-dose ICS, adherent	57.20% of #6	38,410	
**8**	Uncontrolled patients with ≥ 2 exacerbations/year*	47.57% of #7	**18,270**	
**9**	**Uncontrolled patients with severe asthma**	0.04% of #1	**21,649**	[39]
**10**	**Average number uncontrolled patients** **with severe asthma**	Average of #8 and #9	**19,960**	Calculated

*ICS* inhaled corticosteroids, *ISTAT* italian institute of statistics

*Corresponding to 0.034% of the Italian population, ≥ 12 years (#1)

#### Step 3

**Fig. 3:**
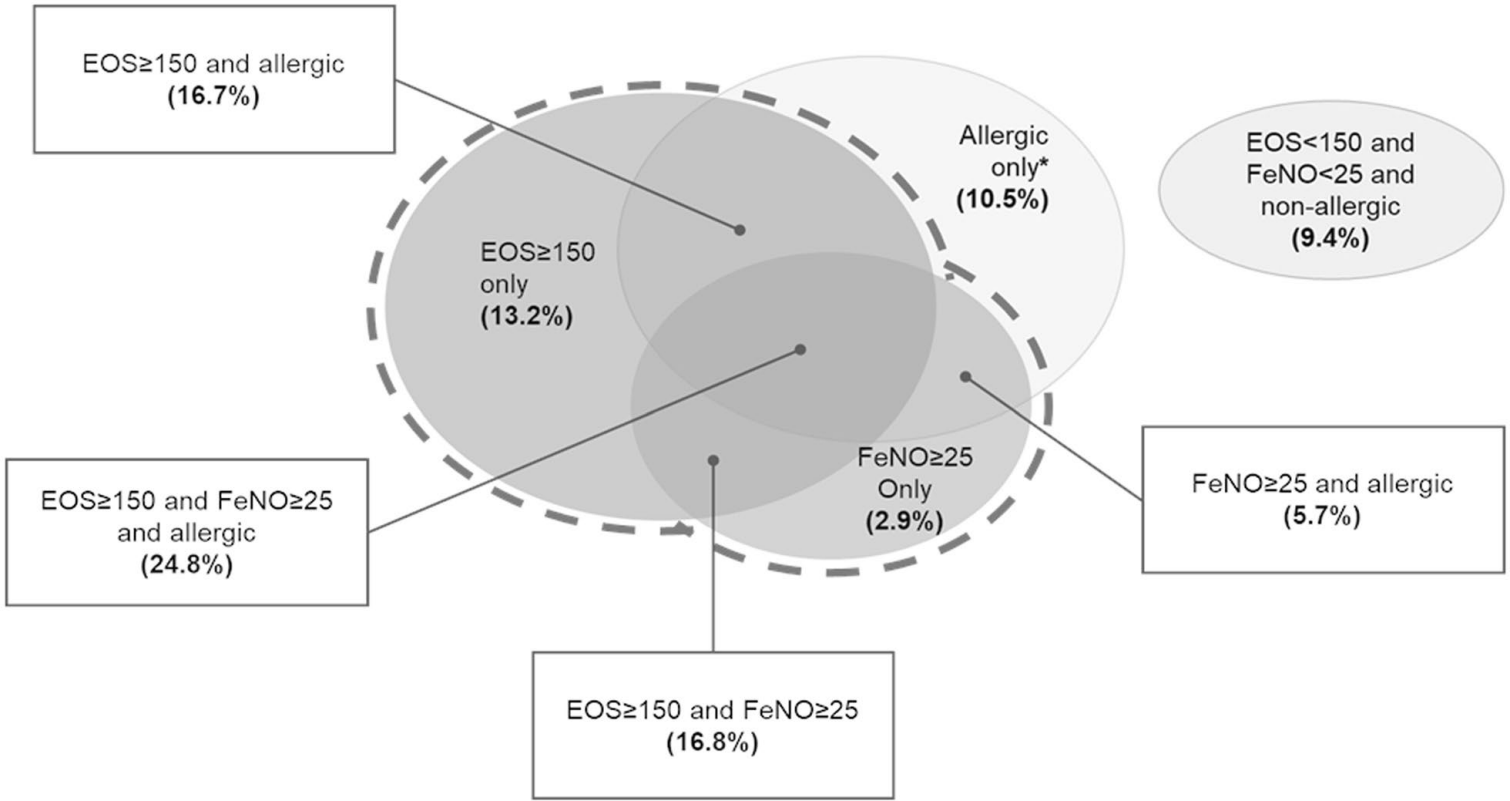
Distribution of patients by Type 2 inflammation biomarkers* [Elaborated from [29]]. In the QUEST trial, 71.5% of the patients had EOS ≥ 150 cells/μL; 50.2% had FeNO ≥ 25 ppb and 57.7% were allergic (IgE ≥ 30 IU/mL). 24.8% of the patient population presented at baseline EOS ≥ 150 cells/μL and FeNO ≥ 25 ppb and IgE ≥ 30 IU/mL. 64.0% of the patient population presented an increase of at least two of the three identified relevant biomarkers at baseline (41.6% had EOS ≥ 150 cells/μL and FeNO ≥ 25 ppb). 26.6% of the patient population presented an increase of only one of the three identified relevant biomarkers at baseline

The third step of the analysis was to stratify the severe uncontrolled asthma population by biomarker levels. Stratification of Type 2 asthma by biomarker status is important to identify patients with blood EOS < 150 cells/μL or FeNO < 25 ppb, who must be excluded from patient estimation. For this purpose, the QUEST trial (a randomized, double-blind, placebo-controlled, parallel-group trial assessing the efficacy of dupilumab in patients with uncontrolled moderate-to-severe asthma; NCT02414854) was considered an appropriate source to evaluate the distribution of patients by Type 2 inflammation biomarkers: EOS, FeNO and IgE [29]. Figure 3 provides a graphical representation of patient distribution, by biomarker status, at baseline as per QUEST trial.

In general, the three largest groups were: (i) patients with increase of both EOS and FeNO levels (EOS ≥ 150 cells/μL and FeNO ≥ 25 ppb) and allergic (IgE ≥ 30 IU/mL; 24.8% of patient population); (ii) patients with increase of both EOS and FeNO levels (EOS ≥ 150 cells/μL and FeNO ≥ 25 ppb) and non-allergic (IgE < 30 IU/mL; 16.8% of patient population); (iii) patients with EOS ≥ 150 cells/μL, FeNO < 25 ppb and allergic (IgE ≥ 30 IU/mL; 16.7% of patient population).

#### Step 4

According to baseline patients’ characteristics of the QUEST trial, two patient groups were considered non-eligible to dupilumab: (i) patients with EOS < 150 cells/μL, FeNO < 25 ppb and allergic (IgE ≥ 30 IU/mL; 10.5% of patient population); (ii) patients with EOS < 150 cells/μL, FeNO < 25 ppb and non-allergic (IgE < 30 IU/mL; 9.4% of patient population). In conclusion, the QUEST trial indicates that 80.1% of patients with severe uncontrolled Type 2 asthma (100.0%-10.5%-9.4%) would have raised blood EOS and/or raised FeNO and would be then eligible for dupilumab treatment.

## Results

The input data collected through the 4-step approach were used to calculate the number of dupilumab-eligible patients. According to these estimates, about 3.3 million asthmatic subjects live in Italy (Table 1; line 2). If findings of the analysis conducted in Veneto are reproportioned to Italy, we would expect that ~ 330 thousand patients with asthma receive at least one spirometry per year (10% of the overall population), and almost half of them (~ 156 thousand) receive some treatment with ICS. A small, but non-negligible proportion of these patients, between 0.034% and 0.04%, are affected by uncontrolled asthma, resulting into N = 18,270–21,649 patients (Table 1, lines 8 and 9, respectively).

On average, the number of patients with QUEST-like characteristics would be N = 19,960. This number accounts for ~ 6% of all asthmatic patients who receive some regular follow up (i.e. patients with spirometry) and ~ 13% of all actively treated asthmatic patients (i.e. patients receiving ICS).

Table 2 provides stratification of the N = 19,960 Italian patients, if their characteristics were comparable to those of the QUEST patients at baseline. Most patients (N = 12,774; 64.0% = groups 1,2,3,4) would have ≥ 2 raised biomarkers and almost one out of four patients (N = 4950 in total) would have EOS, FeNO and IgE raised levels simultaneously. Dupilumab-eligible patients would be N = 15,988, corresponding to 80.1% of the target population.

**Table 2:** Estimation of patients with severe uncontrolled asthma in Italy, by dupilumab eligibility [29]

#	Patient subgroup°	Proportion of pts (%)	EOS ≥ 150 cells/μL	FeNO ≥ 25 ppb	IgE ≥ 30 IU/mL*	Dupilumab-eligible	Number of eligible pts (N)
*-*	*All patients*	*100.0%*	*n/a*	*n/a*	*n/a*	*n/a*	*19,960*
1	**EOS ≥ 150 cells/μL** and **FeNO ≥ 25 ppb** and IgE ≥ 30 IU/mL	24.8%	✔	✔	✓	Yes	4950
2	**EOS ≥ 150 cells/μL** and FeNO < 25 ppb and IgE ≥ 30 IU/mL	16.7%	✔		✓	Yes	3333
3	EOS < 150 cells/μL and **FeNO ≥ 25 ppb** and IgE ≥ 30 IU/mL	5.7%		✔	✓	Yes	1138
4	**EOS ≥ 150 cells/μL** and **FeNO ≥ 25 ppb** and IgE < 30 IU/mL	16.8%	✔	✔		Yes	3353
5	**EOS ≥ 150 cells/μL** and FeNO < 25 ppb and IgE < 30 IU/mL	13.2%	✔			Yes	2635
6	EOS < 150 cells/μL and **FeNO ≥ 25 ppb** and IgE < 30 IU/mL	2.9%		✔		Yes	579
7	EOS < 150 cells/μL and FeNO < 25 ppb and IgE ≥ 30 IU/mL	(10.5%)			✓	No	(2096)
8	EOS < 150 cells/μL and FeNO < 25 ppb and IgE < 30 IU/mL	(9.4%)				No	(1876)
-	*All dupilumab-eligible patients*	*80.1%*	*n/a*	*n/a*	*n/a*	*Yes*	*15,988*

*EOS* eosinophils, *FeNO* fractional exhaled nitric oxide, *IgE* immunoglobulin E, *n/a* not available, *pts* patients

°Biomarkers of interest (EOS ≥ 150 cells/μL and FeNO ≥ 25 ppb) are indicated in bold. *IgE biomarker was evaluated in the QUEST trial; however, IgE alone is not a biomarker to define eligibility for dupilumab. (dupilumab is indicated in Type 2 asthma patients, characterized by raised blood eosinophils and/or raised fractional exhaled nitric oxide, regardless of IgE levels)

**Fig. 4:**
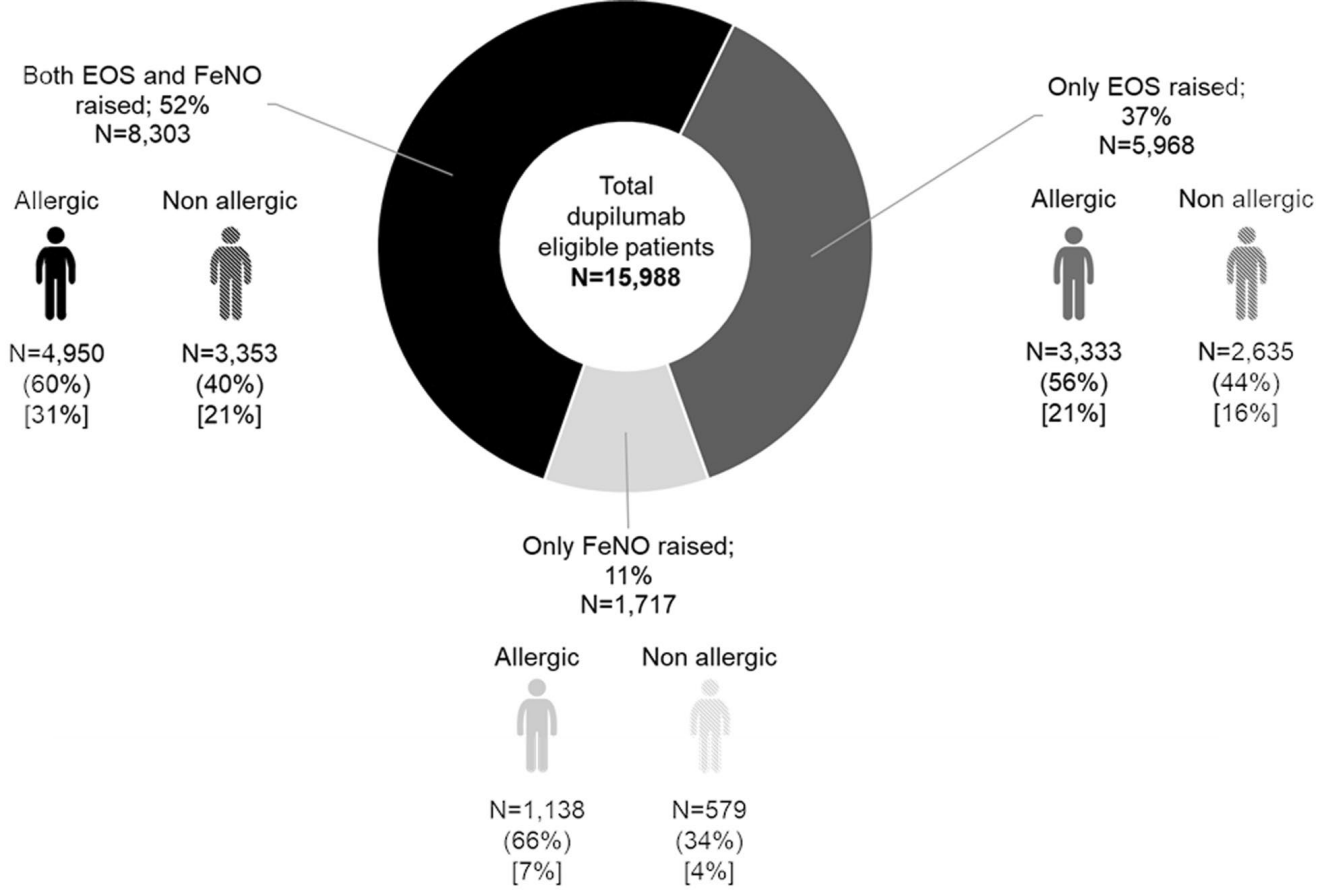
Overview of estimated dupilumab-eligible patient population, in Italy

Finally, Fig. 4 provides an overview of the dupilumab-eligible Italian patient population by EOS and FeNO levels, with further stratification by presence of allergic disease (IgE ≥ 30 IU/mL). More than half eligible patients (N = 8303) present raised levels of both biomarkers, while the large majority (89.3%) has at least an increase of EOS levels (N = 14,271). Increased FeNO levels without increased EOS are observed less frequently (N = 1717; 10.7% of the eligible population). In all the three subgroups, there are more allergic (i.e. IgE ≥ 30 IU/mL) than non-allergic patients (59.6% in the both EOS and FeNO raised group; 55.9% in the only EOS raised group; 66.3% in the only FeNO group). In total, 58.9% of the dupilumab-eligible patient population would also have concomitantly high IgE levels (N = 9421).

## Discussion

The present analysis is an attempt to estimate the epidemiological burden of asthma, and to characterize patient population by severity, type of inflammation and expected distribution of biomarker levels in severe patients. We used data from the QUEST trial to estimate the number of patients with raised EOS and/or raised FeNO to identify the subgroup of patients that would be eligible for dupilumab treatment. The recent administrative database analyses [39, 41] were considered valid tools to estimate the general prevalence of asthma and the proportion of severe cases in Italy. However, a large difference between the registry-based and the administrative-based prevalence rates exists. While the general prevalence of asthma among children and adults is about 6%–7% [23], only ~ 2% of this patient population has an exemption code for asthma [41], meaning that the remaining 4%–5% of patients have very mild forms of asthma and do not consume the same high level of healthcare resources on a regular basis. Interestingly, the two administrative database analyses estimated a similar proportion of patients with severe uncontrolled asthma (i.e. patients with exacerbations requiring hospital care and/or specialized follow-up): 0.04% in the Pedrini et al. study [39] and 0.034% in the Veneto study [41].

Finally, these analyses showed that patients with severe uncontrolled asthma would be ~ 6% of all asthmatic patients who receive some regular follow up (i.e. patients with spirometry) and ~ 13% of all actively treated asthmatic patients (i.e. patients receiving ICS). These figures are quite consistent with published literature, concluding that 5%–10% of patients have a severe form of asthma which is refractory to conventional steroid medical therapy [9].

The QUEST study [29] was used as source to stratify patients with uncontrolled asthma by biomarker levels since it is the only RCT that recruited severe asthmatic patients regardless of minimum baseline blood EOS count or other Type 2 inflammation biomarkers. We chose to use a clinical trial source, rather than a local publication (e.g. the analysis conducted by Heffler et al. [40]), because to our knowledge, QUEST is the only large clinical study providing a complete overview of asthmatic patients subpopulations by Type 2 single biomarker level (i.e. raised EOS, FeNO or IgE) and by grouped biomarkers (e.g. patients with both increased EOS and FeNO level; patients with increased EOS and normal FeNO level, etc.).

Patient distributions, by individual biomarker levels at baseline, were consistent in the QUEST [29] and the SANI (Severe Asthma Network in Italy) cohorts. SANI is an Italian National Registry, promoted by GINA Italy—SIAAIC (Italian Society of Allergy, Asthma and Clinical Immunology) and SIP/IRS (Italian Respiratory Society), which enrolled patients with severe asthma in a real life setting [44]. For instance, the proportion of patients with EOS ≥ 150 cells/μL was 71.5% in the QUEST trial (Table 2), and 79.8% in the SANI registry, thus confirming EOS as the most prevalent biomarker among uncontrolled asthma patients. With regards of FeNO, 50.2% of patients had raised levels (FeNO ≥ 25 ppb) in the QUEST trial, and 50.1% of patients in the SANI registry. In both studies, high proportions of patients were found to have comorbidities such as atopic and nasal polyposis or chronic rhinosinusitis. In the QUEST study, 23.0% of patients had nasal polyposis, 10.3% had atopic dermatitis and 68.6% had allergic rhinitis [29]. In the SANI study, 42.6%, 9.6% and 44.6% of patients had respectively nasal polyposis, atopic dermatitis and allergic rhinitis [40].

According to our estimates, about 20 thousand patients with severe uncontrolled asthma live in Italy, and about 16 thousand patients (~ 80%) have a Type 2 endotype with raised EOS and/or FeNO, who would make them potentially eligible to dupilumab. Whilst most of dupilumab-eligible patients demonstrate raised EOS level (N = 14,271, 89.3%), there is still a non-negligible number of patients (N = 1717, 10.7% of the eligible population) that have increased FeNO levels without increased EOS. This suggests the importance of testing both the biomarkers, during the diagnosis of Type 2 asthma and in the disease follow-up. Indeed, some patients may have clinically uncontrolled asthma but the biomarker expression could be suppressed by pharmacological treatment [1]. Moreover, according to GINA guidelines, the efficacy of the severe asthma therapy must be evaluated through disease control (reduction in exacerbation, improvement in lung function, oral corticosteroids use reduction, etc.) rather than reduction of biomarkers levels [45].

Plausibly, there are a few methodological limitations in this analysis, that might affect validity of findings and increase uncertainty of the estimates. First, we used administrative database analyses, rather than clinical registry data, to estimate the overall population with uncontrolled asthma. This might lead to underestimation of patients, as administrative databases do not identify patients: (i) seeking private care; (ii) with intermittent disease, who would temporarily be “out of the system” but would likely reappear later in time. It is estimated that 5%–10% of all asthma patients develop severe refractory disease [46, 47]. The estimate presented here represents the patient population with uncontrolled severe asthma, despite adequate therapy compliance. Therefore, there is a risk that such methodology would include the population with the highest medical need and exclude milder forms that will exacerbate later. However, we believe that such an estimate would still be more precise and accurate than registry-based assessments, which are based on much smaller sample sizes and would tend to overestimate eligible patients. A second limitation of the analysis is the use of clinical trial data (i.e. QUEST) to stratify the asthma population by biomarker levels. QUEST data were preferred over real-practice Italian data due to their completeness but might not be representative of the local situation. However, some of the possible comparisons between QUEST and SANI cohorts showed a good level of consistency between the two sources, suggesting that our choice of using QUEST data was methodologically acceptable.

According to GINA guidelines, clinical assessment of severe asthma is a crucial step for disease characterization, as it ensures identification of patients requiring urgent healthcare interventions and highly effective treatments. Biomarker assessment is essential to identify the expression of Type 2 inflammation. According to GINA guidelines and prescribing conditions defined by regulatory agencies, the increase of at least one of the severe Type 2 asthma biomarkers would justify the use of biological therapy. However, in clinical practice, it is important to understand the complexity of the inflammatory mechanisms of Type 2 inflammation for each patient, to choose the treatment with the highest probability of acting against the different Type 2 inflammatory components expressed or co-expressed by the patient. Testing for more biomarkers simultaneously, is strongly recommended during baseline patient assessment and disease follow-up, to identify the possible targets of biological therapy (EOS, IgE, FeNO). As a matter of fact, patients with normal EOS but increased FeNO have similar disease severity (i.e. same forced expiratory volume -FEV1-, exacerbation rate and asthma control questionnaire score -ACQ-) as patients with raised EOS and normal FeNO, or patients with both raised EOS and FeNO. In a post-hoc analysis of the QUEST study [36], the efficacy of dupilumab was assessed by biomarker subgroups, as defined by GINA. Baseline blood EOS count and FeNO levels clearly showed that disease severity was similar in all sub-populations (only EOS ≥ 150 cells/μL, only FeNO ≥ 20 ppb, both EOS ≥ 150 cells/μL and FeNO ≥ 20 ppb) at baseline [48]. Therefore, testing different biomarkers simultaneously during disease follow-up is strongly recommended, to monitor the different inflammatory components of Type 2 inflammation and evaluate adjustment of the biological treatment.

## Conclusions

In conclusion, it was possible to estimate the number of dupilumab-eligible patients in Italy, using data on clinical assessment and biomarker testing from local studies and QUEST trial (EOS and/or FeNO, N = 15,988, 80.1% of patients with severe uncontrolled asthma). There is a strong rationale for simultaneously biomarker testing (including FeNO) during diagnosis and diseases follow-up. With relatively low-cost tests, physicians can estimate the number of patients with severe asthma with Type 2 inflammation (Type 2 asthma), stratify them by phenotypes (eosinophilic, allergic, or mixed), identify the optimal treatment strategy and prescribe biologic therapy appropriately.

## Supplementary Information

**Additional file1 :Table S3.** Codes considered for patient inclusion in the Veneto study [41].

## Data Availability

All data generated or analysed during this study are included in this published article.

## Abbreviations

ACQ: Asthma control questionnaire score
AHR: Airway hyperresponsiveness
ARCo: Accounting and reporting console
ATC: Anatomical therapeutic chemical
EMA: European medicines agency
EOS: Eosinophils
FeNO: Fraction of exhaled nitric oxide
FEV: Forced expiratory volume
GINA: Global initiative for asthma
ICD: International classification of diseases
ICS: Inhaled corticosteroids
IgE: Immunoglobulin-E
IL: Interleukin
ILC: Innate lymphoid cells
iNO: Inducible nitric oxide
ISTAT: Italian institute of statistics
LABA: Long-acting Β2-adrenoreceptor agonists
LTRAs: Cysteinyl-leukotriene type 1 receptor antagonists
n/a: Not available
NO: Nitric oxide
pts: Patients
RCT: Randomized Controlled Trial
SANI: Severe Asthma Network In Italy
SIAAIC: Italian society of allergy, asthma and clinical immunology
SIP/IRS: Italian respiratory society
Th2: T-Helper cell type 2
