# Supplementary material for: Defining type 2 asthma and patients eligible for dupilumab in Italy: a biomarker-based analysis
Source: Clin Mol Allergy. 2021 May 21;19:5. doi: 10.1186/s12948-021-00146-9 (PMC8140446; doi:10.1186/s12948-021-00146-9)
Supplement: Supplementary file 1 — Additional file1 :Table S3. Codes considered for patient inclusion in the Veneto study [41]. [file 12948_2021_146_MOESM1_ESM.docx]

# Appendix

**Table 3. Codes considered for patient inclusion in the Veneto study [41].**

| **Inclusion criteria** | **Code type** | **Code** |
| --- | --- | --- |
| Asthma | Exemption code | 007 |
| Spirometry | Specialist ambulatory codes | 89.37, 89.38 |
| ICS | ATC codes | R03BA, R03AK |
| LABA | ATC codes | R03AC12, R03AC13, R03AC14, R03AC18, R03AK06, R03AK07, R03CC12, R03CC13, R03CC90 |
| Theophylline | ATC codes | R03DA04 |
| High-dose ICS treatment | ATC codes | R03DC |
| Inpatient admission for exacerbation | ICD-9 | 493 |
| Corticosteroids | ATC codes | H02 |

ATC: Anatomical Therapeutic Chemical; ICD: International Classification of Diseases; ICS: Inhaled Corticosteroids; LABA: Long-Acting Beta-Agonists.
